# Alterations of Serum Uric Acid Level and Gut Microbiota After Roux-en-Y Gastric Bypass and Sleeve Gastrectomy in a Hyperuricemic Rat Model

**DOI:** 10.1007/s11695-019-04328-y

**Published:** 2020-03-02

**Authors:** Cunlong Lu, Yu Li, Long Li, Ying Kong, Tuo Shi, Hai Xiao, Shougen Cao, Houxin Zhu, Zequn Li, Yanbing Zhou

**Affiliations:** 1 Department of General Surgery, People’s Hospital of Ju Xian, Rizhao, 276500 Shandong China; 2 Department of Gastrointestinal Surgery, The Affiliated Hospital of Qingdao University, Qingdao, 266000 Shandong China; 3 Department of Gastrointestinal Surgery, Weihai Central Hospital, Weihai, 264400 Shandong China; 4 Department of Gastrointestinal Surgery, Jining No.1 People’s Hospital, No.6 Jiankang Road, Central District, Jining City, Shandong Province China; 5 Department of Cardiovascular Surgery, Baoji Central Hospital, Baoji, Shanxi Province China

**Keywords:** Roux-en-Y gastric bypass, Sleeve gastrectomy, Serum uric acid, Gut microbiota, Lipopolysaccharides, Xanthine oxidase

## Abstract

**Background:**

The objective of this study was to observe alterations of serum uric acid (SUA) level and gut microbiota after Roux-en-Y gastric bypass (RYGB) and sleeve gastrectomy (SG) surgery in a hyperuricemic rat model.

**Method:**

We performed Roux-en-Y gastric bypass (RYGB) and sleeve gastrectomy (SG) surgery in a hyperuricemic rat model. Serum uric acid (UA), xanthine oxidase (XO) activity, IL-6, TNF-α and lipopolysaccharide (LPS) level changes, and 16S rDNA of gut microbiota were analyzed.

**Results:**

After the surgery, the RYGB and SG procedures significantly reduced body weight, serum UA, IL-6, TNF-α and LPS levels, and XO activity. In addition, the RYGB and SG procedures altered the diversity and taxonomic composition of the gut microbiota. Compared with Sham group, RYGB and SG procedures were enriched in the abundance of phylum *Verrucomicrobia* and species *Akkermansia muciniphila*, while the species *Escherichia coli* was reduced.

**Discussion:**

We here concluded that bariatric surgery-induced weight loss and resolution of inflammatory remarkers as well as changes of gut microbiota may be responsible for the reduced XO activity and SUA level. To have a better understanding of the underlying mechanism of UA metabolism following bariatric surgery, further research is needed.

## Introduction

Hyperuricemia (HUA), an abnormality in uric acid (UA) metabolism, which results in the increased serum uric acid (SUA), has become an important issue worldwide and is linked to gout and metabolic diseases, including hypertension, cardiovascular disease, and diabetes [1–3]. The increased SUA in human is mainly due to overproduction of UA from hepatic metabolism by the key enzyme xanthine oxidase (XO) or decreased excretion from the kidney or gut [2]. Therefore, the management of hyperuricemia includes decreased production or increased excretion of UA. Lifestyle management such as weight reduction and dietary modification has less significant SUA-lowering effects [4–6]. However, bariatric surgery could significantly lower SUA level in patients with severe obesity [7–9]. One study has demonstrated that SG-induced SUA reduction may be mediated by reduced XO activity expressed in the white adipose tissues [10]. In addition, other factors may also affect XO activity, such as inflammatory markers IL-6, TNF-α, and LPS [11, 12]. Increased blood circulation of LPS level has been demonstrated to be the result of intestinal microbial imbalance, inducing chronic inflammation along with increased XO activity [13]. Exposure to LPS in mice has been reported to result in XO expression in several organs and tissues; thus, alterations in gut microbiota and LPS exposure may lead to changes in XO expression [11]. A recent study has reported in a hyperuricemic mouse model showing decreased amount of *bifidobacteria* and *lactobacilli*, while increased serum UA level, XO activity, and LPS level [14].

In the current study, we established a hyperuricemic rat model, after that, we performed RYGB and SG surgery and observed alterations in SUA level and gut microbiota. We hypothesize that (1) an increased serum IL-6, TNF-α LPS, and XO activity and alterations in gut microbiota accompanied with increased SUA level in the hyperuricemia rat model; (2) RYGB and SG surgery may change these parameters and influence SUA level.

## Materials and Methods

### Animals

Male Wistar rats (weighing 280–300 g) were purchased from Qingdao Laboratory Animal Co. Ltd. (Qingdao, China) and were housed in the specific pathogen-free (SPF) house including 12-h light/dark cycles, a stable room temperature and relative humidity, and they had free access to their diets and tap water. All animal treatments were approved by the Affiliated Hospital of Qingdao University Ethics Committee. The institutional guidelines for the care and use of laboratory animals were followed throughout the study.

Rats were acclimatized for 1 week animals and randomly divided into two groups: the hyperuricemic model group (*n* = 30) was established by feeding on high-purine diet (10% yeast food and water ad libitum) and intragastrical administration of adenine (0.1 g/kg/day) for 3 weeks, the control group (*n* = 10) fed on laboratory standard chow and intragastrical administration of saline (1 ml/100 g) simultaneously. Three weeks later, the hyperuricemic model group was randomly assigned to three subgroups: RYGB (*n* = 10), SG (*n* = 10), and Sham group (*n* = 10). The randomization was according to the random numeral table method. After the assignment, different operations were performed separately and related parameters were examined.

### Surgical Interventions

Before the surgical operation, the animals were fasted overnight with water available ad libitum. Ten percent chloral hydrate at the dose of 3 ml/kg was used for anesthesia administered by intraperitoneal injection. The RYGB and SG operations were performed as previously described [15]. Three days after the surgery, all the surgical rats were still provided laboratory high-purine food and water ad libitum and the control group remained fed on laboratory standard chow and water ad libitum.

### Sleeve Gastrectomy Procedure

Sleeve gastrectomy procedure included (1) an approximate 4-cm abdominal incision was made; (2) the greater curvature of the stomach (approximately 80% of the gastric volume) was removed; (3) the remaining section was closed using 5–0 sutures in a continuous manner to form the gastric sleeve.

### Roux-en-Y Gastric Bypass Procedure

Roux-en-Y gastric bypass procedure involved (1) an approximate 4-cm midline abdominal incision was created; (2) Treitz ligament was identified and measured approximately 10 cm distant from the duodenum within the jejunum and was transected to form the Roux limb and biliary pancreatic limb; (3) identified the visible white ridge that is the edge of the forestomach and the glandular stomach and along which transected and sewed by 5–0 silk sutures in a continuous manner to form a gastric pouch; (4) a 5-mm incision was made on the lateral wall of the forestomach and the antimesenteric wall of the jejunum respectively, followed by a gastrojejunostomy and jejunojejunostomy was created using 6–0 Prolene suture with side-to-side anastomosis.

### Sham Surgery

For the sham surgery, a 5-mm anterior gastrostomy and a jejunostomy were performed, followed by reanastomosis using 6–0 sutures.

### Blood Sample Collection and Analysis

Preoperative (0 week) and postoperative 2-, 4-, 6-, and 8-week blood samples were obtained and centrifuged 3000 rpm for 10 min for the serum samples. The serum samples were stored at a temperature of − 80 °C, and subsequently prepared for further examination. Serum level of uric acid was examined using Automatic biochemical analyzer (Hitachi 7600A, Japan). Serum lipopolysaccharides (LPS), inflammatory markers interleukin-6 (IL-6), tumor necrosis factor-α (TNF-α), and xanthine oxidase (XO) levels were measured by using ELISA kit method (InvitrogenTM of Thermo Fisher Scientific corporation, Shanghai, China).

### Fecal Sample Collection, Processing, and Analysis

Fecal samples were collected with sterile EP tubes and immediately stored at 80 °C, which were later analyzed using 16S rRNA gene high-throughput sequencing technology. Microbial genomic DNA of fecal samples from rats were extracted using (QIAamp) stool DNA extraction kit according to the manufacturer’s instruction. 16S rDNA variable region V3-V4 was amplified by polymerase chain reaction (PCR) using the forward primer (515F:GTGCCAGCMGCCGCGGTAA) and reverse primers (806R: GGACTACHVGGGTWTCTAAT). A unique 10-bp barcode was used to tag each sample following with each forward primer. DNA amplification of microbial 16S rRNA gene was performed using the KAPA SYBR FAST qPCR Kit (Kapa Biosystems, Boston, MA, USA). The amplification system included 25 μl Failsafe Premix F (Epicentre Biotechnologies, Madison, WI, USA), 0.4 μM each primer, 2.5 U of Ex Taq DNA polymerase (Takara, Dalian, China), and 1–2 μl DNA template in a total volume of 50 μl. PCR was run at 98 °Cfor 5 min, followed by 27 cycles of 98 °Cfor 30 s, 50 °Cfor 30 s, and 72 °Cfor 30 s with a final extension at 72 °C for 5 min. All samples were amplified in triplicate, pooled, and purified using the QIAquick PCR purification kit (Qiagen, Valencia, CA, USA). Amplicons were sequenced using Illumina MiSeq, and after that, the pyrosequencing data processing was further performed using QIIME (Version1.17). Raw pyrosequencing data were processed using Mothur (Version 1.31.2, http://www.mothur.org/) to obtain unique reads. After filtering the low-quality reads, the remaining high-quality clean data were analyzed later. Clean tags were connected through the overlap of reads by Ribosomal Database Project (RDP) Classifer v.2.2 to taxonomically classify Operational taxonomic units (OTU) representative sequences in the following databases: Greengene V201305; RDP (Version 2.2). OTUs were denoted at the level of 97% similarity (cutoff value was set to 0.6) using UPARSE (Version 7.0.1090) based on the clean tags. Chimera generated from the amplicons was removed from the OTU reads using UCHIME (Version 4.2.40).

### Statistical Analysis

All the data were expressed as the mean ± standard deviation (SD). Serum UA, LPS, IL-6, TNF-α, and XO levels were evaluated by using one-way analysis of variance (ANOVA) followed by Bonferroni post hoc comparison. Pearson association and linear regression analysis were used when analyzing the relationship between variables. Statistical analyses were performed using IBM SPSS 19.0 version software. A value of *P <* 0.05 was considered statistically significant.

The Chao and ACE index was assessed to show the richness of gut microbiota. The nonparametric richness estimator observed_species and Shannon index were used to evaluate the Alpha diversity with Mothur (Version 1.31.2), and dilution curve was generated using the R program (Version 2.15.3). The Beta diversity was estimated by unweighted UniFrac distances using QIIME (Version1.17). Significant difference analyses were calculated using Metastats (http://metastats.cbcb.umd.edu/), and *P* values corrected in R soft package (Version 2.15.3). The Kruskal-Wallis rank sum test was employed to evaluate the differences between each group. Fecal bacterial analysis was performed by BGI technology (Shenzhen, China).

## Results

### Effect of RYGB and SG Surgery on Body Weight and Amounts of Food Intake

**Fig. 1:**
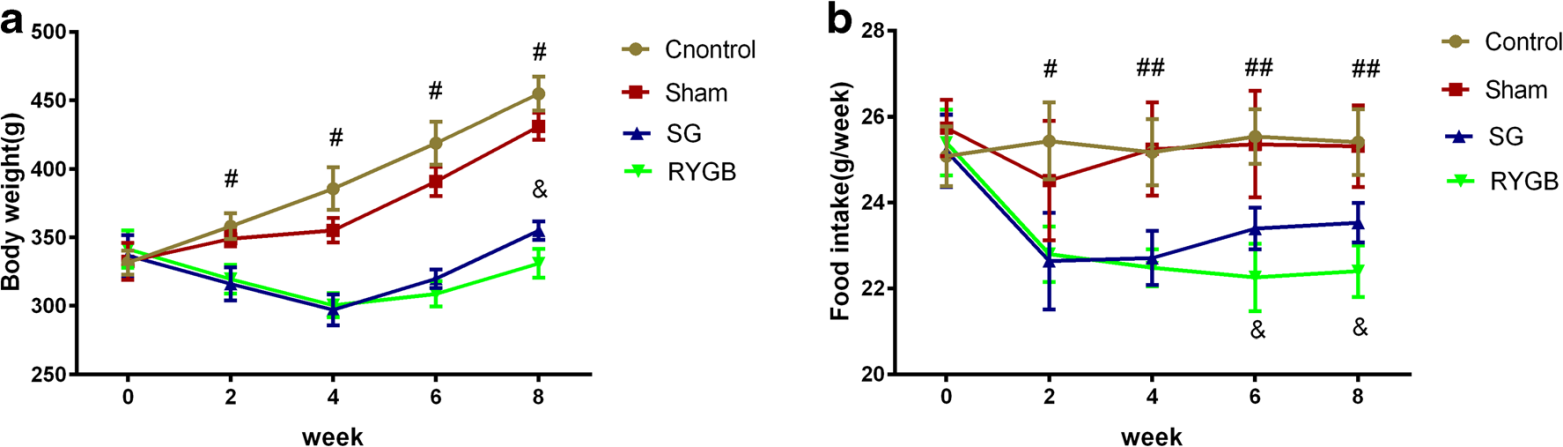
Effects of RYGB and SG on body weight (**a**) and food intake (**b**). ^#^*P <* 0.01 RYGB or SG vs. Sham. ^##^*P <* 0.001 RYGB or SG vs. Sham. ^&^*P <* 0.01 RYGB vs. Sham

Two rats in the RYGB group died of anastomotic fistula and were excluded. The remaining of other rats all survived throughout the study until 8 weeks postoperation. Before the surgery, the mean body weight of all rats among the four groups exhibited no statistical difference (*P >* 0.05). The preoperative and postoperative changes in the body weight of rats are shown in Fig. 1a. The body weight of Control and Sham groups showed a sustained increase 2, 4, 6, and 8 weeks after surgical operation. Compared with both the Sham and Control groups after surgery, RYGB and SG resulted in sustained weight loss during the observed stages of 2 and 4 weeks postoperation (*P* < 0.01). Although the body weight of RYGB and SG rats gradually increased 4 weeks after the surgery, they remained lower than those of Sham and Control groups. Prior to the operation, the amounts of food intake of all the rats were not significantly different (*P >* 0.05). Postoperatively at week 2 and 4, compared with Sham and Control groups, RYGB and SG groups considerably induced a reduction in the amounts of food intake as shown in Fig. 1b. SG group gradually increased food intake after 4 weeks, which was higher than that of RYGB group (*P <* 0.05), but was still lower than those of Control and Sham groups (*P* < 0.01).

### Changes in SUA Levels in Different Treatment Groups

**Fig. 2:**
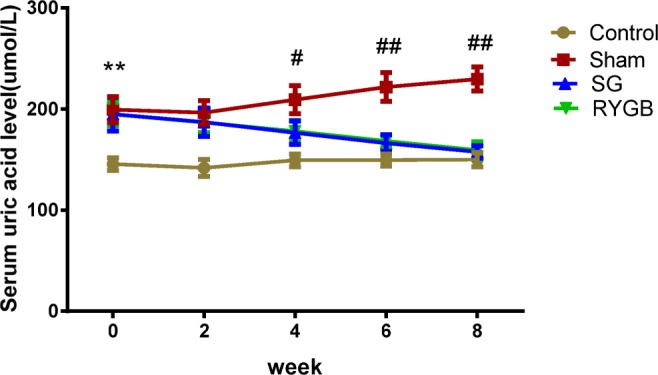
Effects of RYGB and SG on serum uric acid level in hyperuricemic rat model. **P < 0.001 Control vs. Sham. *P < 0.05 RYGB or SG vs. Sham. ^#^*P <* 0.01 RYGB or SG vs. Sham. ^##^*P* < 0.001 RYGB or SG vs. Sham

After 3-week treatment, the mean SUA levels of the hyperuricemic model groups were markedly higher than that of Control group (*P <* 0.001). Among the model groups, no significant difference among the three subgroups (RYGB, SG, Sham) was observed before the surgical procedure (*P >* 0.05). Postoperatively at 4, 6, and 8 weeks, RYGB and SG surgery significantly decreased the SUA levels compared with that in Sham group (*P <* 0.01). Changes of SUA levels before and after surgery are shown in Fig. 2.

### Effects of RYGB and SG on Serum LPS, XO, IL-6, and TNF-α levels

**Fig. 3:**
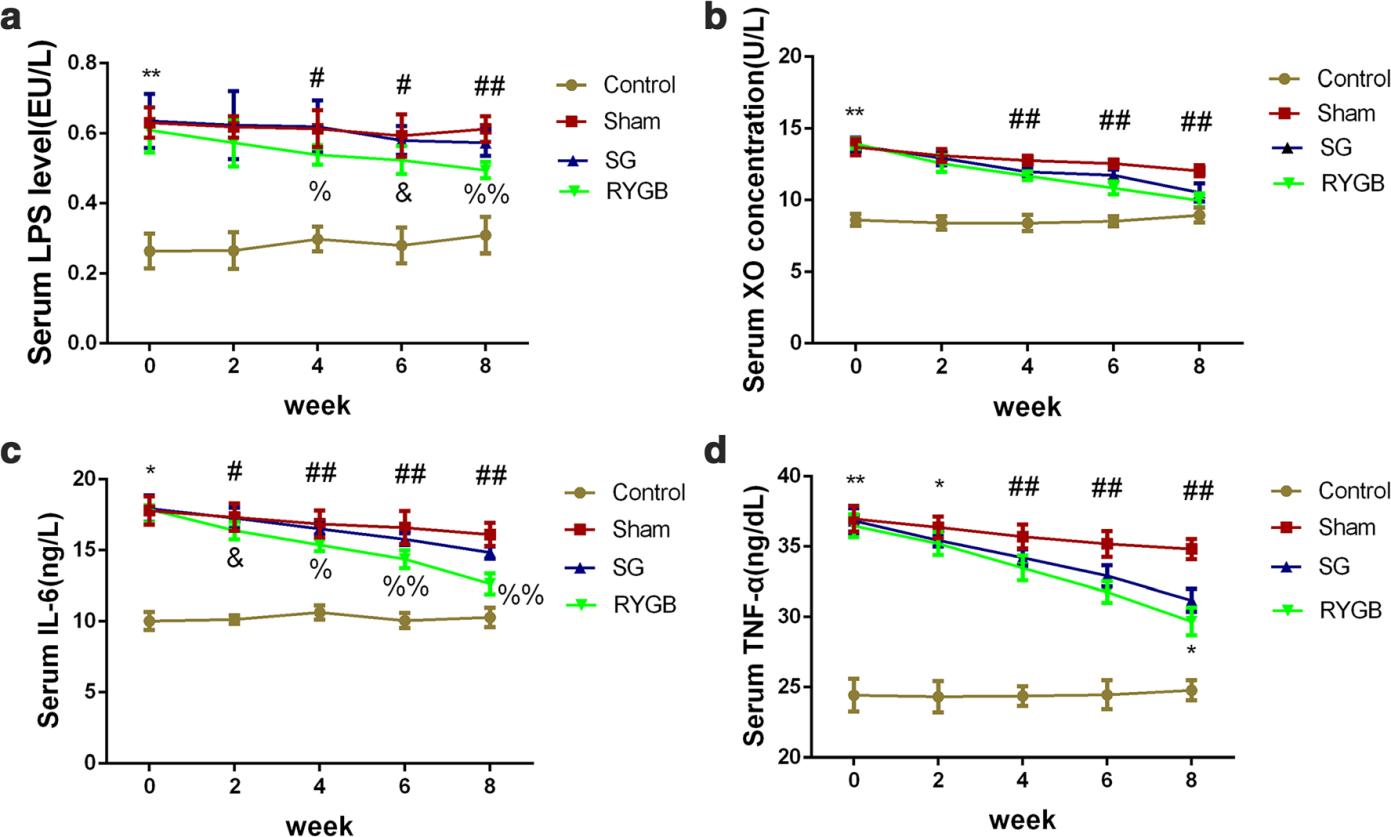
Effects of RYGB and SG on serum LPS (**a**), XO (**b**), IL-6 (**c**), and TNF-α (**d**) in hyperuricemic rat model. **P < 0.001 control vs sham. ^#^*P <* 0.01 RYGB vs. sham. ^%^*P <* 0.01 RYGB vs. SG. ^%%^*P <* 0.001 RYGB vs. SG. *P < 0.05 SG vs. sham. ^##^*P <* 0.001 RYGB or SG vs. sham

Prior to the surgical operation, serum levels of LPS, XO, IL-6, and TNF-α in the hyperuricemic group were significantly higher than those in Control group (*P <* 0.001). Postoperatively, RYGB significantly reduced the LPS level at week 4, 6, and 8 compared with the Sham (*P* < 0.01) and SG (*P* < 0.05) groups. Two weeks after surgery, serum concentration of XO between the Sham and SG groups was not significant, but the RYGB group showed a significant lower XO concentration (*P* < 0.05), and 4 weeks after the surgery, the XO concentration of the RYGB and SG groups was significant lower than that in the Sham group (*P* < 0.001). Two weeks after surgery, the RYGB group significantly reduced serum IL-6 level compared with the Sham and SG groups (*P* < 0.01 vs. Sham; *P* < 0.05 vs. SG), and RYGB surgery significantly lowered serum IL-6 level in comparison with that in the Sham group 4, 6, and 8 weeks after surgery (*P* < 0.001), while SG surgery significantly reduced IL-6 levels compared with the Sham group only observed in week 6 and 8 following surgery. For the TNF-α level, compared with the Sham group, RYGB and SG surgery markedly lowered the serum TNF-α 4, 6, and 8 weeks after surgery (*P* < 0.01). Changes in serum LPS, XO, IL-6, and TNF-α concentrations before and after surgery are shown in Fig. 3a, b, c, and d.

### Correlation of Changes of SUA with Other Changed Variables

In order to investigate whether changes of SUA (delta SUA) were associated with other clinical outcomes, the relationship between delta SUA and other parameters was analyzed performed by using multiple regression analysis. Changes of all variables were calculated as baseline minus 8-week follow-up. The pooled RYGB and SG cohorts showed that delta SUA following RYGB and SG significantly correlated with delta-body weight (standardized *β* = 0.794, *P =* 0.036). In addition, we also found delta-LPS (standardized *β* = 0.08, *P* = 0.047), TNF-α (standardized *β* = 2.265, *P* = 0.00), and IL-6 (standardized *β* = 1.850, *P* = 0.00) were significantly associated with delta-XO following RYGB and SG.

### Gene Richness and Diversity Changes in the Gut Microbiota After RYGB and SG

**Fig. 4:**
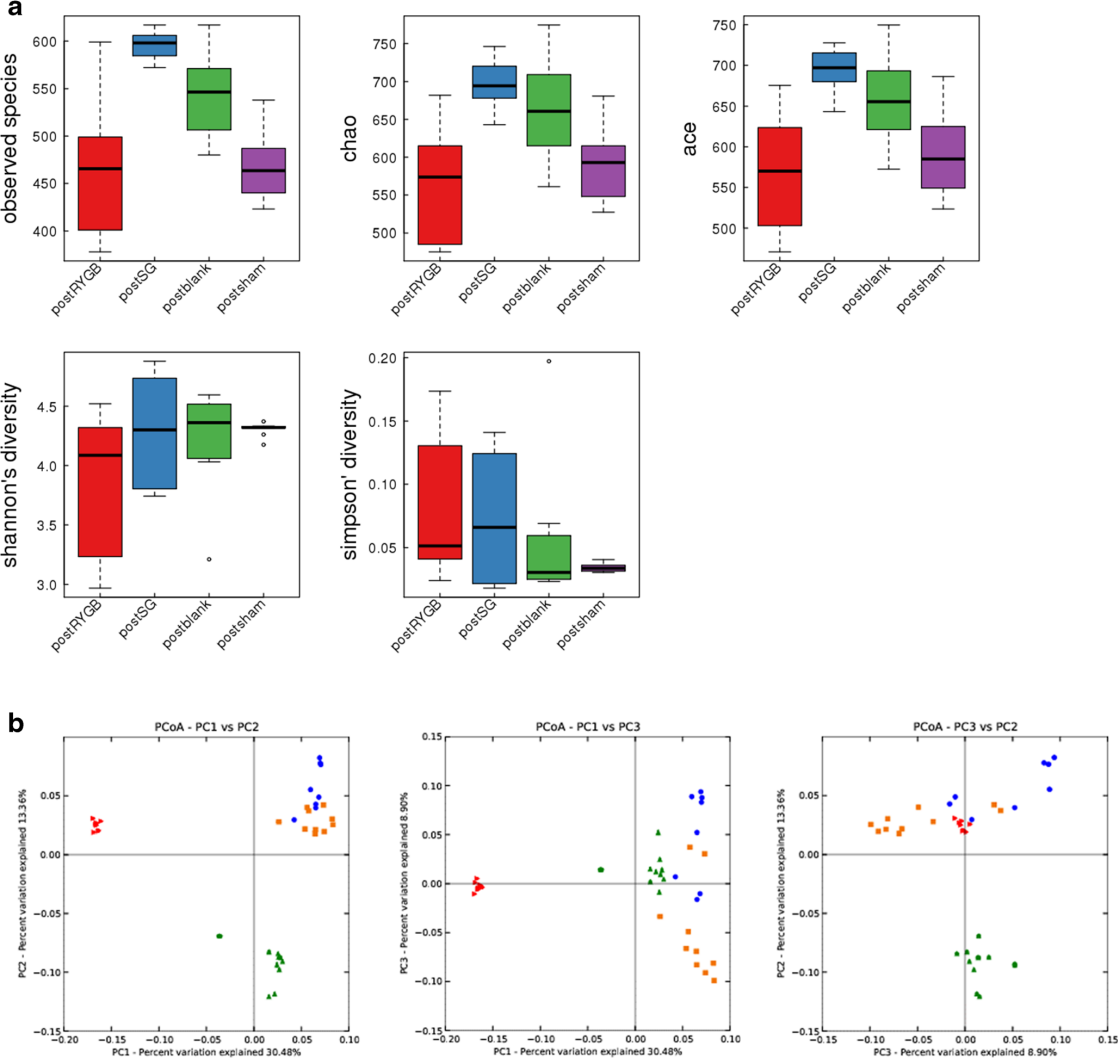
**a** Community richness and alpha diversity alteration after RYGB and SG surgery. **b** Principal coordinate analysis (PCoA) based on unweighted UniFrac from the 38 fecal samples of each group. Each dot represents a sample, and different colors denote the samples collected from the different groups. PC1: first principal coordinate, percent variation explained 30.48%; PC2: second principal coordinate, percent variation explained 13.36%; PC3: third principal coordinate, percent variation explained 8.90%

The richness of gut microbiome estimated by observed species, Chao and ACE indices showed that compared with RYGB and Sham treatments, SG surgery increased the richness (*P* < 0.01). The results of the Shannon index which estimated the alpha diversity of gut microbiota suggested that no significant difference was observed among the four groups (*P* > 0.05) (Fig. 4a). For the beta diversity, unweighted UniFrace-based principal coordinate analysis from 38 fecal samples after surgery revealed separated clusters among the RYGB, Sham, and Control groups, but the SG group showed a closer cluster to the Control group (Fig. 4b).

### RYGB and SG Altered the Taxonomic Composition of Gut Microbiota in the Hyperuricemic Rat Model

**Fig. 5:**
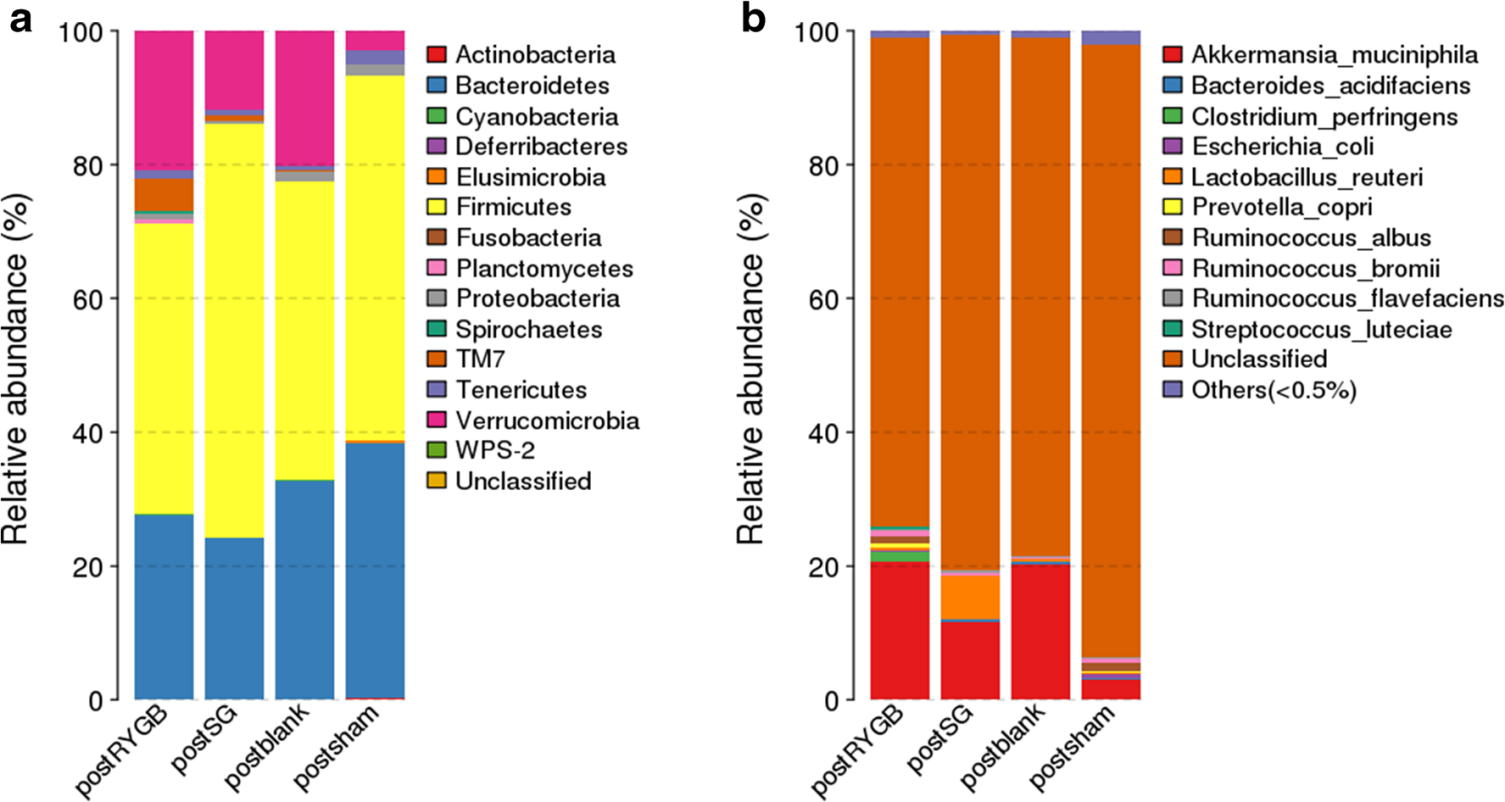
Relative abundance of the dominant phyla (**a**) and species (**b**) of each group. Significant difference analyses were calculated using Metastats (http://metastats.cbcb.umd.edu/), and *P* values corrected in R (Version 2.15.3). PostRYGB, postSG, and postsham represented the relative abundance of fecal microbiota sampled 8 weeks after surgery, while the postblank group represented the relative abundance of fecal microbiota of control group in the 8-week follow-up

Postoperatively, at the endpoint of week 8 during the experimental process, fecal samples from the RYGB, SG, Sham, and Control groups analyzed by 16S rDNA sequencing using Illumina MiSeq showed that at the phylum levels, *Firmicutes*, *Bacteriodetes*, *Verrucomicrobia*, and *Proteobacteria* accounted for almost 90% of the gut microflora. The relative abundance of phylum *Proteobacteria* in the RYGB (1.51 ± 0.83%) and Sham (1.62 ± 1.43%) group was higher than that in SG (0.96 ± 0.30%) and Control (0.43 ± 0.29%) groups (*P* < 0.01). The relative abundance of *Verrucomicrobia* in the RYGB (19.84 ± 11.68%) and SG (17.21 ± 18.10%) groups was much higher than that in the Sham group (3.05 ± 0.98%) (*P* < 0.01) (Fig. 5a).

At the species level, the percentage of *Akkermansia muciniphila* (*A. muciniphila*) in the RYGB (19.84 ± 11.68%) and SG (17.21 ± 18.10%) groups was significantly higher than that in the Sham group (3.05 ± 0.98%) (*P* < 0.01), while the relative abundance of *Escherichia coli* in the RYGB (0.018 ± 0.025%) and SG (0.064 ± 0.028%) groups was significantly lower than that in the Sham group (0.66 ± 0.56%) (*P* < 0.01) (Fig. 5b).

## Discussion

In the current study, we investigated the changes of body weight, serum IL-6, TNF-α, LPS levels, and XO activity following bariatric surgery. Indeed, we observed significant decrease in SUA, serum inflammatory remarkers, and XO activity, accompanied by reduction in body weight. We also performed the correlation between SUA and XO activity and other indices and we interestingly found weight loss was associated with changes in SUA. In addition, we also found that changes in IL-6, TNF-α, and LPS were associated with XO activity. Although no significant correlation between XO activity and SUA was found, we still observed a decrease in XO activity, which may partially resulted in the decreased SUA. A recent study has documented that weight loss by meal replacement therapy resulted in reduction in XO activity, while with no significant decrease in SUA level [6], while Andreas Oberbach et al. showed a reduced SUA level and expression of XO in adipose tissue accompanied by weight loss following SG surgery [10]. Thus, we hypothesize that alteration of gastrointestinal (GI) and XO activity may be the key influencing factor. It is known that GI changes induced by RYGB and SG surgery influence GI hormones, relative abundance of gut microbiota, and chronic inflammatory status [16, 17], which may interfere the UA metabolism by reducing the expression of XO activity in some tissues.

Obesity is demonstrated to be associated with increased XO activity, UA levels, and cytokines. Inflammatory cytokines including IL-6 and TNF-α have been reported to be a regulator for the XO expression [6]. IL-6 is reported to play an important role in the regulation of XO expression because the repressor proteins binding to the promoter region of the XO gene can be inactivated by IL-6 [18, 19]. Studies have demonstrated that weight loss resulted in the reduction of inflammatory cytokine levels, such as IL-6 and TNF-α [20, 21], accompanied by decreased XO and SUA level [6, 22]. Therefore, we hypothesized that the bariatric surgery resulted in body weight loss accompanied by decreased inflammatory cytokines. In the current study, the concentrations of blood circulation of TNF-α and IL-6 were decreased after RYGB and SG surgery. Additionally, we observed delta-TNF-α and IL-6 were associated with delta-XO, our results demonstrated that decreased TNF-α and IL-6 following RYGB and SG may induced decreased expression of XO, which resulted in decreased production of UA.

Another factor that modulates the blood XO activity is the component of gram-negative bacteria cell wall LPS [11]. A recent study reported that animals exposed to LPS challenge exhibited increased plasma XO activity [13]; therefore, alterations of gut microbiota and an increased level of circulatory LPS may be the responsible for the changes in XO concentration in our study. Furthermore, an increased expression of XO at both the activity and gene levels was found after the administration of inflammatory cytokines [12] in vitro and after LPS administration in vivo [11]. Cao et al. [14] found that a significant reduction in the percentage of *Bifidobacteria* and *Lactobacillis*, with an increase in the level of serum UA, XO activity, and LPS in the hyperuricemic C57BL/6 mice compared with the mice without hyperuricemia, and supplementation with the two probiotic strains *Bifidobacteria* and *Lactobacillis* reversed the changes of gut microbiota and decreased the SUA level and XO activity. Thus, regulating the gut microbiota may be a target for the treatment of hyperuricemia. The current study showed that the hyperuricemic rat model significantly increased serum LPS level, which may be the results of imbalance of gut microbiota. LPS causes an increase in the expression of XO activity, may be partially responsible for the increased SUA level. Studies have observed that bariatric surgery significantly reduced plasma LPS level, with resolution of insulin resistance and T2DM [23, 24]. Indeed, we also observed significant correlation between delta-LPS and delta-XO. Therefore, we supposed that bariatric surgery may regulate the gut microbiota to reduce serum LPS level and cytokine levels, leading to reduced XO expression and decreased SUA level.

Studies have also shown that modification of gut microbiota by bariatric surgery is associated with improved metabolic status [25–27]. However, the impact of the RYGB and SG procedures on the changes of hyperuricemia-induced gut microbiota remains unknown. We previously found that relative abundance of phylum *Proteobacteria* in hyperuricemic rat model was much higher than that in control group, and the increased phylum *Proteobacteria* was mainly due to the increased species *Escherichia coli* (data not shown). In the current study, we found that RYGB and SG surgery altered the taxonomic composition of the gut microbiota in the hyperuricemic rat model. The relative abundance of phylum *Proteobacteria* in Control and SG groups was lower than those in RYGB and Sham groups. Unfortunately, unlike other studies suggesting that RYGB resulted in increased relative abundance of *Proteobacteria* [27–29], we did not find significant difference in the phylum *Proteobacteria* between RYGB and Sham group. We surmised that RYGB surgery in our study may not significantly affect the phylum *Proteobacteria* and may be due to the reduction in the relative abundance of species *Escherichia coli* following RYGB surgery. However, interestingly, we documented that the RYGB and SG groups were enriched in the relative abundance of phylum *Verrucomicrobia*. The increased abundance of *A. muciniphila* species almost contributes to the increase in the relative abundance of phylum *Verrucomicrobia*. Unlike the changes of phylum *Proteobacteria* after RYGB and SG surgery, the above two surgery showed the similar tendency as the relative abundance of *A. muciniphila.* Interestingly, RYGB and SG surgery also lead to the similar decreased trend of SUA level. Increased *A. muciniphila*, a mucin-degrading bacterium, was suggested to be a contributor to the maintenance of gut health, glucose homoeostasis, and decreased adipose tissue inflammation and increased gut integrity [30–33]. Evidence has also demonstrated that bariatric surgery increased *A. muciniphila* in humans and mice [34, 35]. Therefore, we surmised that changes of *A. muciniphila* after RYGB and SG surgery may be associated with decreased SUA level. In addition, we found that RYGB and SG surgery reduced the relative abundance of *Escherichia coli* compared with Sham group. LPS, a material that derived from the cell wall of *Escherichia coli* that can induce the increased expression of XO, has been reported [11]. The reduced relative abundance of *Escherichia coli* after RYGB and SG group demonstrated that the alteration in *Escherichia coli* may be a factor that regulates XO expression by influencing LPS level.

 There are several limitations in the current study. Firstly, this study was only a pilot experiment in rat model, furthermore, due to the species difference between rodents and human beings, the results of our study may not reflect the actual fact; therefore, more work will be done to investigate in larger human cohorts. Secondly, although we detected changes of serum XO level following RYGB and SG surgery, we observed no correlation between changes of SUA and serum XO level. However, we found that weight loss following RYGB and SG surgery correlated with decreased SUA as well as XO level. Since the XO activity mainly expressed in the liver, adipose, and intestinal tissues, weight loss may mediated the reduction of expression of XO in adipose following bariatric surgery. In addition, we found that changes of XO activity were correlated with changes of LPS, IL-6, and TNF-α following RYGB and SG surgery. Therefore, we concluded that weight reduction, alteration of gut microbiota, and decreased inflammatory tones following RYGB and SG surgery may mediate the reduction of XO activity and SUA level. However, due to the complex of regulation of UA metabolism and the specific mechanisms of bariatric surgery-induced amelioration of metabolism remains unclear, therefore, further investigations are warranted. Although we found that some groups of intestinal microbes increased or decreased following RYGB and SG surgery, whether these changes have an effect on the UA metabolism remains unknown. Transfer of fecal microbiota from RYGB-operated or SG-operated animals to germ-free hyperuricemia animals and the effects of fecal microbiota transplantation (FMT) on SUA level will be investigated in our next study.
